# Human Leukocyte Antigens and Epstein–Barr Virus-Associated Nasopharyngeal Carcinoma: Old Associations Offer New Clues into the Role of Immunity in Infection-Associated Cancers

**DOI:** 10.3389/fonc.2013.00299

**Published:** 2013-12-09

**Authors:** Wen-Hui Su, Allan Hildesheim, Yu-Sun Chang

**Affiliations:** ^1^Department of Biomedical Sciences, Graduate Institute of Biomedical Sciences, College of Medicine, Chang Gung University, Taoyuan, Taiwan; ^2^Chang Gung Molecular Medicine Research Center, Chang Gung University, Taoyuan, Taiwan; ^3^Infections and Immunoepidemiology Branch, Division of Cancer Epidemiology and Genetics, National Cancer Institute, Bethesda, MD, USA

**Keywords:** genome-wide association study, nasopharyngeal carcinoma, HLA antigens, EBV, infection associated cancers

## Abstract

Nasopharyngeal carcinoma (NPC) is an Epstein–Barr virus (EBV) associated tumor. In addition to EBV, host genetic factors are believed to be important determinants of NPC risk. Of all genes studies to date, human leukocyte antigen (HLA) genes have shown the most consistent evidence for association with NPC, both from candidate-gene studies and genome-wide association studies (GWAS). In this report we summarize results from recent studies that evaluated the association between HLA and NPC, and discuss whether findings reflect direct causal associations for HLA genes and/or indirect associations that mark causal associations with other genes in the gene-dense major histocompatibility (MHC) region where HLA resides. We also compare GWAS results across cancer sites for which strong hits in the MHC region were observed to generate new hypotheses regarding the role of HLA genes in the development of EBV-associated cancers such as NPC. Of note, we report that MHC associations for EBV-associated cancers (NPC, EBV+ Hodgkin lymphoma) are driven by HLA class I genes. In contrast, MHC associations for other viral-associated cancers (cervical cancer, hepatocellular carcinoma) or other hematopoetic cancers (EBV− Hodgkin lymphoma, leukemia, non-Hodgkin lymphomas) are driven by HLA class II genes, and those for other solid tumors with less clear links to infections (lung, testicular, prostate cancers) are driven by non-HLA genes in the MHC region. Future studies should aim to better understand these patterns.

## Introduction

Nasopharyngeal carcinoma (NPC) is an epithelial malignancy that is common in regions of Southeast Asia and the Mediterranean Basin. The age-adjusted incidence rate of this tumor in Southern China, for example, is 25–30 cases per 100,000 person years, which is approximately 50 times higher than what is observed in the Western world (1–3). Infection with Epstein–Barr virus (EBV) is believed to be a near necessary factor for the development of NPC (3, 4). EBV is a ubiquitous infection that typically occurs in early life, establishes lifelong latent infection in B-lymphocytes, and periodically reactivates in the epithelial compartment of the pharynx (5). Since EBV infection is common and NPC is rare, it is widely agreed that other environmental and genetic factors are important determinants of NPC risk. With respect to host genetic factors associated with NPC, human leukocyte antigens (HLA) have been proposed to be important, given their central role in presentation of viral antigens to the immune system (6).

The HLA genes comprise a family of highly polymorphic genes located within the major histocompatibility complex (MHC) on chromosome 6p21.3. An association between HLA genes and NPC was first proposed by Simons and colleagues (7). Since that initial report, the association between HLA genes and NPC has been confirmed in over 100 candidate-gene-based association studies (3, 8, 9). More recently, three independent genome-wide association studies (GWAS) of NPC consistently identified SNPs within the MHC region (where HLA genes are located) as having the strongest evidence for association with NPC (10–12).

In this review, we summarize recent findings regarding the association between HLA genes and NPC susceptibility. We then discuss whether the associations observed in the gene-rich MHC region, where strong linkage disequilibrium (LD) patterns are observed, are driven only by HLA genes or whether other non-HLA genes in the region might also be involved. Finally, we compare GWAS results across cancer sites for which strong hits in the MHC region were observed, to generate new hypotheses regarding the role of HLA genes in the development of EBV-associated cancers such as NPC.

## Human Leukocyte Antigen Associations with Nasopharyngeal Carcinoma

Human leukocyte antigen genes are located within the MHC region on chromosome 6p21. The MHC region is a gene-dense region (>150 genes) that also exhibits some of the strongest LD patterns within the human genome (13). These features of the MHC region make studies of HLA-cancer association particularly challenging because it is often difficult to determine whether reported associations are causal and/or reflect LD with other genes in this region. Nonetheless, there is a strong biological *a priori* for a causal association between HLA genes and NPC, given that HLA molecules are central to the presentation of viral peptides to cytotoxic and helper immune cells, and that infection with EBV is ubiquitously associated with the development of NPC. Of relevance to this review, there are three classical HLA class I genes, namely, *HLA-A*, *HLA-B*, and *HLA-C*, and three clusters of classical HLA class II genes, namely, *HLA-DR*, *HLA-DP*, and *HLA-DQ* that have been evaluated for their association with NPC. Both HLA class I and class II molecules can bind peptides through their peptide recognition groove and present peptides to T cells. However, HLA class I and II genes differ with respect to the types of cells in which they are expressed and the types of immune cells they regulate. HLA class I molecules are expressed on most nucleated cells and typically present foreign peptides to cytotoxic T cells. HLA class II molecules have a more restricted expression pattern being normally expressed on B-lymphocytes and antigen-presenting cells that typically present foreign peptides to helper T cells (13).

As alluded to in the Introduction, the association between HLA and NPC was first proposed in 1974 and since that time over 100 candidate-gene association studies have consistently reported associations between HLA alleles/haplotypes and NPC (3, 8, 9). More specifically, studies have reproducibly reported associations with NPC for the following HLA class I alleles: HLA-A*0207 (risk allele in LD with HLA-B*4601), HLA-A*1101 (protective allele in LD with HLA-B*13), and HLA-B*5801 (risk allele in LD with HLA-A*3303) (9, 14). Linkage analyses also suggested that HLA-A and HLA-B genes are associated with the development of NPC (15–17). Other HLA genes, including *HLA-C* and HLA class II genes, showed less consistent findings across studies (9).

**Figure 1:**
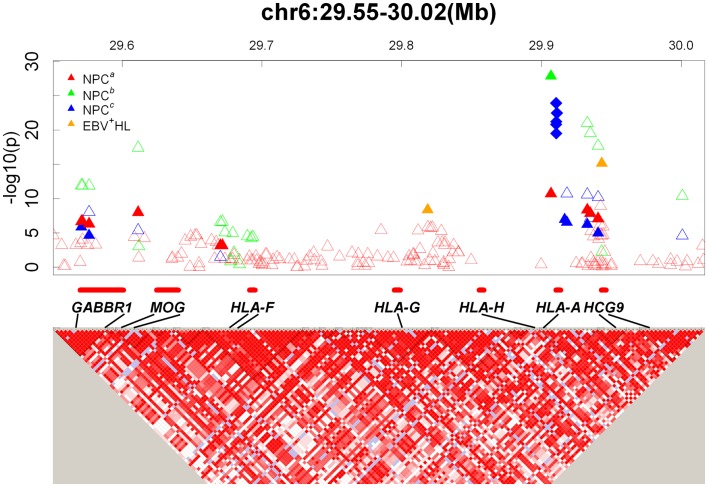
**GWAS results map in the HLA-A locus**. Top: Triangles and diamonds dots indicate the *P* values from different GWASs on a minus logarithmic scale according to the chromosome location of the SNPs. Different studies are labeled with different colors. Red: NPC^a^ (10); Green: NPC^b^ (11); Blue: NPC^c^ (12); Orange: EBV^+^ HL (21). The solid triangles indicate significant SNPs reported in GWASs. The hollow triangles indicate other SNPs listed in the supplementary documents. The solid diamonds indicate significant amino acids located within the HLA genes (12). Bottom: Detailed LD structure depicted in HaploView using control samples from the NPC^a^ GWAS (10). The increasing intensities of red represent lower *D′* values.

With the advent of technologies to interrogate the entire genome to better understand the genetic architecture of complex diseases (18), it has become possible to evaluate HLA-NPC associations in the context of genetic associations in other regions of the genome. To date, four NPC GWAS have been reported (10–12, 19). Of these four studies, three reported the strongest hits in the MHC region, where HLA genes reside (10–12) and only one GWAS (the smallest with a total of 110 NPC cases and 260 controls in the discovery phase) did not report genome-wide significant hits in the MHC region (19). The largest NPC GWAS to date from Guangdong, China (3,477 and 6,570 individuals in discovery and replication phases, respectively) reported their strongest finding to be located in HLA-A (rs2860580; *P*_GWAS_ = 1.34 × 10^−28^, *P*_combined_ = 4.88 × 10^−67^, OR = 0.58; Figure 1, NPC^b^) (11). The strongest reported associations for the Taiwan (562 and 2,275 individuals in discovery and replication phases, respectively) (10) and Guangxi, China (1,043 and 985 individuals in discovery and replication phases, respectively) (12). NPC GWAS were also located in HLA-A (Taiwan GWAS: rs2517713, *P*_GWAS_ = 3.56 × 10^−8^, *P*_combined_ = 3.90 × 10^−20^, OR = 1.88, Figure 1, NPC^a^; Guangxi, China GWAS: rs417162, *P*_GWAS_ = 1.13 × 10^−7^, *P*_combined_ = 1.05 × 10^−11^, OR = 0.63, Figure 1, NPC^c^). It is noteworthy to point out that rs2860580 and rs2517713 are in complete LD, and that the Guangxi, China GWAS (12) also observed strong evidence for association for rs2517713 (*P*_GWAS_ = 3.03 × 10^−7^, *P*_combined_ = 1.63 × 10^−11^, OR = 0.60, Figure 1, NPC^c^). While significant associations were reported for SNPs in the HLA-B/C and HLA-DR/DQ regions in the Guangdong, China NPC GWAS (rs2894207; *P*_GWAS_ = 1.22 × 10^−16^, *P*_combined_ = 3.42 × 10^−33^, OR = 0.61; rs28421666; *P*_GWAS_ = 3.54 × 10^−9^, *P*_combined_ = 2.49 × 10^−18^, OR = 0.67, respectively) (11) they were not as strong as those observed for SNPs located in HLA-A. Furthermore, in the Guangxi NPC GWAS (12), where high resolution HLA genotyping was performed, multivariate analyses suggested that associations were driven by HLA-A*1101 and that presence of glutamine at amino acid position 62 of the HLA-A gene (which marks HLA-A*11) was strongly associated with NPC risk providing a biological basis for the SNP-based associations reported.

Taken together, findings from both candidate-gene-based studies and GWAS have consistently demonstrated associations between HLA genes and NPC. The strongest evidence for association has been observed for alleles within HLA class I genes, including HLA-A*0207 (risk allele in LD with HLA-B*4601), HLA-A*1101 (protective allele in LD with HLA-B*13), and HLA-B*5801 (risk allele in LD with HLA-A*3303). The associations are biologically plausible and suggest a direct causal association defined by specific amino acids involved in defining HLA binding specificities.

## Non-Human Leukocyte Antigen Associations in the MHC Region with Nasopharyngeal Carcinoma

In addition to HLA genes, other genes in the MHC region of chromosome 6p21.3 have been found to be associated with NPC. Notably, results from the Taiwan NPC GWAS (10) suggested significant associations for the HLA complex group 9 (*HCG9*) gene (rs9260734, *P*_GWAS_ = 2.49 × 10^−7^, *P*_combined_ = 6.77 × 10^−18^, OR = 1.85), and the gamma-aminobutyric acid (GABA) B receptor 1 (*GABBR1*) gene (rs29232, *P*_GWAS_ = 1.67 × 10^−8^ *P*_combined_ = 8.97 × 10^−17^, OR = 1.67). These associations were also observed in the two NPC GWAS from China (11, 12), suggesting that the associations observed are real and not driven by chance. Given the strong LD patterns observed in the MHC region, however, the issue of whether these associations implicate new genes involved in NPC or mark one or more of the HLA associations discussed above is still an open question.

HCG9 is a non-protein coding gene located within 30 kb of the *HLA-A* gene. Given its close proximity to *HLA-A* and lack of known function, it is hypothesized that SNPs within this gene reported to be associated with NPC in recently published GWAS reflect LD with causal polymorphisms within *HLA-A*. In fact, multivariate analyses of data from the Taiwan (10) and two China (11, 12) GWAS suggested that hits within the HCG9 region are not independent of the *HLA-A* findings. Furthermore, a recent pooled analysis of two NPC case-control studies in Taiwan that jointly evaluated HLA and HCG9 confirmed that the HCG9 signal observed in the original Taiwan GWAS is likely to be driven by HLA rather than an independent signal (20).

In contrast to *HCG9*, *GABBR1* encodes a G protein-coupled receptor that forms a heterodimer with GABAB receptor 2, thereby triggering downstream signaling events in the proliferation, differentiation, and migration of cancer cells. This known biological function provides some *a priori* support for a possible functional role of *GABBR1* in cancer development. Following the initial Taiwan GWAS finding, sequencing of full-length *GABBR1* genes, including the rs29232 region, was performed in 37 NPC cases with high-risk haplotypes and 48 controls. No germ line mutations were detected and most of the novel association signals either failed to be validated or did not reach the statistical significance of rs29232 (10). However, in this same study expression of the GABBR1 protein in NPC tissues was also evaluated using immunohistochemical staining and the intensity of the GABBR1 signal in tumor cells was significantly higher than that detected in adjacent normal epithelial cells (*P* < 0.001) (10). This finding provides additional support for a potential functional role for *GABBR1* in the etiology of NPC.

Statistical approaches have also been employed in an attempt to determine whether GWAS findings in *HLA-A* and *GABBR1* represent distinct, independent associations, or whether they represent a single association marked by SNPs in both gene regions. Results from these efforts have been mixed, however, multivariate logistic regression analysis of the Taiwan GWAS data indicated that the *GABBR1* signal (rs29232) remained significant after controlling for the effect of *HLA-A* GWAS SNPs and sequence-based *HLA-A* alleles, namely, HLA-A*0207/0215N or HLA-A*110101/0121N (10). Similar efforts from the two China GWAS, however, suggested that the *GABBR1* effect was largely driven by the *HLA-A* SNPs/alleles (11, 12). An analysis of pooled data from two NPC case-control studies conducted in Taiwan that jointly evaluated *HLA-A* and *GABBR1* reported that while the effect of *GABBR1* was attenuated by adjustment for *HLA-A* alleles, a significant effects for *GABBR1* (rs29232) remained after adjustment for *HLA-A* and that an independent effect of *GABBR1* could therefore not be ruled out (20).

In summary, while *HLA* genes, and *HLA-A* in particular, likely explain much of the signals observed from NPC GWAS in the MHC region on chromosome 6p21.3, the possibility that other genes in the region, such as *GABBR1*, might also be causally involved in NPC cannot be discarded at this time.

## Human Leukocyte Antigen Associations in Other Cancer Genome-Wide Association Studies

Close to 180 cancer GWASs have been published (NHGRI GWAS catalog^1^). We reasoned that examination of findings from the MHC region across these GWAS might provide interesting clues into the link between HLA genes and infection-associated cancers such as NPC. We therefore summarized findings within the MHC region from cancer GWAS available through the NHGRI GWAS catalog^1^. To organize our review, we classified cancers into mutually exclusive groups as follows: EBV-associated cancers [including NPC, classical Hodgkins lymphoma (HL), and gastric cancer], other infection-related cancers [including cervical cancer and hepatocellular carcinoma (HCC)], other immune-related (hematopoetic) tumors [including various subtypes of non-Hodgkin lymphomas (NHL)], and other solid tumors (including lung, testicular, and prostate cancers). While these groupings are somewhat arbitrary (e.g., we grouped gastric cancers with EBV-associated cancers despite its strong link with *H. pylori* infection since approximately 8% of gastric cancers are known to be EBV-positive and the focus of this review is on NPC, another EBV-associated cancer), we believe that they provide a useful rubric for summarizing findings across a diverse set of cancers. We focused our review on the MHC region alone (defined as chr6: 29 –33 Mb) and considered as significant SNPs with a reported *P*-value for association <5 × 10^−7^. Findings are summarized in Table 1.

**Table 1:** **Cancer GWAS significant signals located in chromosome 6p21 MHC region**.

Disease	Year	No. of cases/No. of controls	HLA class I genes		HLA class II genes			Other MHC genes	Reference
			*HLA-A*	*HLA-B/C*	HLA-DR	HLA-DQ	HLA-DP		
EBV-related tumors^a^
Nasopharyngeal carcinoma	2009	111/260							(19)
Nasopharyngeal carcinoma	2009	277/285	rs2517713					*GABBR1*, *HCG9*, *HLA-F*	(10)
			rs2975042						
Nasopharyngeal carcinoma	2010	1,583/1,894	rs2860580	rs2894207		rs28421666		*GABBR1*, *HCG9*	(11)
Nasopharyngeal carcinoma	2012	1,405/1,650	rs417162					*GABBR1*, *HCG9*	(12)
Hodgkin’s lymphoma	2011	589/5,199			rs6903608				(22)
Hodgkin’s lymphoma	2012	1,200/6,417			rs2395185			*MICB*	(21)
EBV-positive Hodgkin’s lymphoma			rs2734986						
EBV-negative Hodgkin’s lymphoma					rs6903608				
Nodular sclerosis Hodgkin’s lymphoma	2011	393/3,315			rs204999 rs9268528				(23)
Other virus-related tumors
Hepatocellular carcinoma	2011	180/271						*C2*	(34)
HCV-related hepatocellular carcinoma	2011	721/2,890				rs9275572		*MICA*	(30)
HBV-related hepatocellular carcinoma	2012	1,538/1,465				rs9272105			(31)
HBV-related hepatocellular carcinoma	2013	1,161/1,353				rs9275319			(32)
HBV-related hepatocellular carcinoma	2013	971/1,938			rs9269081	rs2856718, rs7453920	rs9277535, rs3077	*EHMT2*, *TCF19*	(33)
Cervical Cancer	2013	1,034/3,948			rs9272143^e^	rs9272143^e^	rs3117027	*MICA*	(28)
Cervical Cancer	2013	1,364/3,028					rs4282438		(29)
Other hematopoetic tumors^b^
Follicular lymphoma	2009	189/592			rs6457327				(44)
Follicular lymphoma	2010	681/750				rs10484561		*C6orf15*	(45)
Follicular lymphoma	2011	379/791				rs2647012			(46)
Chronic lymphocytic leukemia	2010	407/296			rs674313	rs9272535			(47)
Chronic lymphocytic leukemia	2012	1,121/3,745						*BAK1*, *IRF4*	(48)
Chronic lymphocytic leukemia	2013	3,100/7,667			rs674313	rs9273012, rs9273363		*IRF4*	(49)
Lymphoma^c^	2013	1,245/2596			rs4530903^e^ rs9268853	rs4530903^e^ rs2647045 rs2621416			(50)
Other solid tumors^d^
Lung cancer	2008	1,952/1,438						*BAG6/APOM*	(51)
Lung cancer	2013	5,510/4,544			rs2395185				(35)
Lung adenocarcinoma	2009	5,739/5,848						*BAG6/APOM*	(52)
Lung adenocarcinoma	2012	1,695/5,333						*BTNL2*	(53)
Testicular germ cell tumor	2009	730/1,435						*BAK1*	(54)
Prostate cancer	2011	6,621/6,939						*CCHCR1*	(55)
Prostate cancer	2013	11,085/11,463						*NOTCH4*	(56)
Multiple cancers^f^	2012	5,368/4,006						*LRFN2*	(27)

*^a^ GWAS study in gastric cancer (25–27) and non-cardia gastric cancer (24) with no significant findings reported in the MHC region*.

*^b^ GWAS studies in acute lymphoblastic leukemia (childhood) (36–39), chronic myeloid leukemia (40), and large B-cell lymphoma (41) with no significant findings reported in the MHC region*.

*^c^ This study included multiple types of lymphoma: 275 follicular non-Hodgkin lymphoma cases, 269 diffuse large B-cell non-Hodgkin lymphoma cases, 198 other non-Hodgkin lymphoma cases, 202 Hodgkin lymphoma cases, and 4,044 controls*.

*^d^ GWAS studies in basal cell carcinoma, bladder cancer, breast cancer, colorectal cancer, endometrial cancer, esophageal cancer, Ewing sarcoma, gallbladder cancer, glioblastoma, glioma, melanoma, multiple myeloma, neuroblastoma, ovarian cancer, ovarian reserve, pancreatic cancer, renal cell carcinoma, small-cell lung cancer, testicular cancer, thyroid cancer, urinary bladder cancer, and Wilms tumor with no significant findings reported in the MHC region (NHGRI GWAS catalog http://www.genome.gov/gwastudies)*.

*^e^ rs9272143 and rs4530903 located between *HLA-DRB1* and *HLA-DQA1**.

*^f^ This study included 2,331 lung cancer cases, 1,006 non-cardia gastric cancer cases, 2,031 esophageal squamous-cell carcinoma cases, and 4,006 Han Chinese controls*.

First, we considered cancers that are known to be linked to EBV infection (Table 1). In addition to NPC (for which nearly 100% of tumors are EBV-associated), we evaluated two cancers for which a proportion of tumors are known to be EBV-associated: HL and gastric cancer. Burkitt lymphoma (BL) was not considered despite its close association with EBV infection because no BL GWAS has been published to date. As discussed in previous sections of this review, results from NPC GWAS indicate that the strongest evidence for an association are with HLA-A, an HLA class I gene. Findings from HL GWAS suggest an interesting pattern. The single GWAS that evaluated EBV(+) and EBV(−) HL separately (21) found evidence for an HLA class I association for EBV(+) HL and for an HLA class II association for EBV(−) HL. The HLA class I association observed for EBV(+) HL is consistent with findings for NPC. The HLA class II association observed for EBV(−) HL is consistent with findings for other hematopoetic cancers and cancers caused by infections other than EBV (discussed further below). A second HL GWAS that did not stratify by EBV status provides evidence for association with HLA class II but failed to observe and association with HLA class I (22). Finally, a GWAS that focused on nodular sclerosis HL, a subtype of HL thought not to be EBV-associated, also reported associations with HLA class II genes only (23). This highlights the importance of stratifying analyses on viral status for this heterogeneous disease. Finally, four gastric cancer GWAS have been published (24–27). None reported evidence for a significant association within the MHC region. It should be noted, however, that <10% of gastric cancers are EBV(+) and so the lack of association observed within the MHC might reflect the lack of stratification on EBV status rather than a true lack of association for EBV(+) gastric cancers. In summary, GWAS of EBV(+) cancers have consistently reported associations in the MHC region, and within the MHC the strongest evidence points to an important role for HLA class I genes.

Next, we evaluated results from GWAS for other infection-associated cancers (Table 1). Of the cancers with proven link to viral infections other than EBV, GWAS have been reported for cervical cancer (linked to human papillomavirus infection) and HCC (linked to hepatitis B and C infections, HBV and HCV respectively). Two cervical cancer GWAS reported the strongest evidence for association within the HLA class II region of the MHC (28, 29). The four HCC GWAS that specifically evaluated HCV-related or HBV-related cancers all reported strong hits within the HLA class II region (30–33). In contrast, the one HCC GWAS that did not stratify by virus status (34) observed evidence for association within the MHC region, but failed to report significant associations within HLA, again highlighting the importance of stratification by viral status for etiologically heterogeneous tumors. Of note, none the four GWAS of gastric cancer, a cancer linked with infection with the bacteria *Helicobacter pylori*, reported significant associations within the MHC region, suggesting differences in the HLA associations observed for bacterial- versus viral-associated cancers. In summary, all GWAS of non-EBV virus infection-associated cancers (HPV, HBV, and HCV) that specifically considered viral status reported evidence for HLA class II associations with disease. This contrasts with EBV-associated cancers, where evidence consistently points to HLA class I involvement.

Finally, we examined GWAS for other hematopoetic and solid tumors (Table 1). Again, clear and consistent patterns emerged. For other hematopoetic tumors, signals observed in the MHC region were consistently located within the HLA class II region or outside of the HLA class I or II regions. In contrasts, for other solid tumors for which significant signals were observed in the MHC region, the observed signals were consistently located outside of the HLA class I or II regions and appeared to involve non-HLA genes. The one exception to this was the HLA class II (rs2395185) association observed for lung cancer in never-smoking woman in Asia (35). It should be noted that, in contrast to observations for infection-associated cancers, significant MHC signals were not always observed for other hematopoetic and solid tumors. For example, amongst hematopoetic tumors, no evidence for significant associations within the MHC region were reported for GWAS of childhood acute lymphoblastic leukemia (36–39), chronic myeloid leukemia (40), and diffuse large B-cell lymphoma (41). For other solid tumors, no evidence for significant associations within the MHC region were reported for GWAS of basal cell carcinoma, bladder cancer, breast cancer, colorectal cancer, endometrial cancer, esophageal cancer, Ewing sarcoma, gallbladder cancer, glioblastoma, glioma, melanoma, multiple myeloma, neuroblastoma, ovarian cancer, ovarian reserve, pancreatic cancer, renal cell carcinoma, small-cell lung cancer, testicular cancer, thyroid cancer, urinary bladder cancer, and Wilms tumor (NHGRI GWAS catalog^1^).

Taken together, the present review of published cancer GWAS suggests that:

1. HLA class I genes are important for EBV-associated cancers.
2. HLA class II genes are important for HPV, HBV, and HCV-associated cancers.
3. HLA class II and/or non-HLA genes in the MHC region explain associations within the MHC for other hematopoetic malignancies, and
4. There is less evidence for HLA (class I or II) involvement in the development of other solid tumors.

Particularly striking was the tendency for EBV-associated cancers to be linked to HLA class I genes, while other cancers had stronger evidence for HLA class II gene associations (HPV, HBV, and HCV-associated cancers; and some hematopoetic malignancies) or for the involvement of non-HLA genes in the MHC region (other solid tumors and some hematopoetic malignancies). We therefore considered further the characteristics of EBV infection that might explain the “predilection” of EBV-associated cancers to be associated with HLA class I genes.

Little is known about the genetic basis for immunological responses to EBV infection, despite the belief that such responses are important mediators of cancer risk. The only published GWAS that evaluated genetic factors associated with antibodies against EBV reported evidence for the involvement of HLA class II genes in EBV seroreactivity, measured as anti-EBV EBNA-1 IgG levels (42). This finding appears at face value to be inconsistent with the strong HLA class I association observed for NPC, but needs to be interpreted with caution since the anti-EBV antibody evaluated (IgG against EBNA-1) is not a good marker of NPC risk. In the future, it will be important to evaluate genetic factors associated with anti-EBV EBNA-1 and VCA IgA responses because (1) EBNA-1 and VCA are the antigens for which strong and consistent associations with risk of NPC development have been observed and (2) IgA responses (rather than IgG responses) are thought to better reflect chronic EBV reactivation at mucosal surfaces believed to be required for NPC development.

One hypothesis to explain the specific association of HLA class I genes with EBV-associated cancers while HPV, HBV, and HCV-associated cancers have more clear associations with HLA class II genes invokes site of infection. Of all infectious agents known to be directly linked to cancer, EBV is the only one that establishes lifelong latency in B-lymphocytes and requires infection of the epithelial compartment during lytic reactivation for viral shedding and transmission. Perhaps HLA class I genes are important mediators of this complex biological lifecycle, although precisely how this might be remains to be established. Of interest to note in this respect is the fact that progression to AIDS among HIV infected individuals has been consistently shown to be associated with HLA class I alleles (43). Since HIV is a virus that, like EBV, establishes lifelong infection in lymphocytes, the commonality of these findings might suggest a parallel HLA-mediated immunological response pathway for these two pathogens that is distinct from that for other infectious agents linked to cancer development.

## Challenges and Opportunities

Human leukocyte antigen genes have long been suggested to be associated with NPC risk. Recent NPC GWAS have confirmed this association and have further indicated that the strongest genetic associations with NPC reside within HLA class I genes, particularly *HLA-A*. This contrasts with what has been observed for other tumors, suggesting a unique relationship between EBV and HLA class I genes and the need for studies to more specifically define how and which host genetic differences in antigen presentation of specific EBV antigens affects the EBV tumorigenesis process by promoting viral escape of host immune surveillance leading to NPC. These studies could provide clues not only about EBV and NPC, but also about the relationship between other oncogenic infectious agents and their respective cancers and on the nature of HLA associations for hematopoetic malignancies.

While challenges remain, given the strong LD patterns observed in the MHC, the large number of genes in this region and the highly polymorphic nature of HLA genes themselves, the prospect of studying diverse populations with distinct HLA patterns and LD structure, and of applying new technologies such as high-throughput sequencing and molecular profiling techniques to elucidate the complex structure of the MHC and its association with NPC and other cancers could lead to better insights into our understanding of the specific mechanisms involved in cancer pathogenesis. This, in turn, could lead to new interventions aimed at interrupting this pathogenic process as a cancer prevention measure.

## Conflict of Interest Statement

The authors declare that the research was conducted in the absence of any commercial or financial relationships that could be construed as a potential conflict of interest.

## Footnotes

^1^http://www.genome.gov/gwastudies
